# Comparative study of the catalytic performance of physically mixed and sequentially utilized γ-alumina and zeolite in methanol-to-propylene reactions

**DOI:** 10.1098/rsos.240469

**Published:** 2024-09-18

**Authors:** Anahita Mortazavi-Manesh, Nasser Safari, Mojtaba Golbodaqi, Shirin Latifi, Mohammad Fatehi Haghighat, Farzad Bahadoran

**Affiliations:** ^1^ Department of Chemistry, Faculty of Chemistry and Petroleum Science, Shahid Beheshti University, GC, Tehran 1983969411, Iran; ^2^ Gas Research Division, Research Institute of Petroleum Industry (RIPI), Tehran 1485733111, Iran

**Keywords:** mesoporous alumina, zeolite, polyethylene glycol, facile and green method, catalytic performance, methanol-to-propylene

## Abstract

This study aimed to investigate the catalytic performance of H-ZSM-5 zeolite compared with physically mixed and sequentially used synthesized γ-alumina and zeolite in the methanol-to-propylene (MTP) reaction. A facile, green and cost-effective method was first applied to prepare a mesoporous γ-Al_2_O_3_ support using a combination of sol–gel and hydrothermal methods via a few consecutive steps. This process was carried out using aluminium nitrate and polyethylene glycol with different molecular weights as non-ionic surfactants. X-ray diffraction, transmission electon microscopy, thermogravimetric analysis, ammonia temperature programmed desorption and Brunauer–Emmett–Teller analysis were then used to characterize the prepared γ-Al_2_O_3_ catalyst. Afterwards, the catalytic activity of the commercial H-ZSM-5 zeolite (Si/Al = 200) and the effect of the presence of the γ-alumina physically mixed and unmixed with the zeolite were also researched in the MTP reaction. Accordingly, methanol conversion and product selectivity were monitored via gas chromatography. The physically mixed mesoporous γ-Al_2_O_3_ and H-ZSM-5 zeolite exhibited the highest catalytic activity in terms of both conversion and selectivity at 400°C. To our knowledge, this research represents the first documented use of γ-alumina and zeolite simultaneously as catalysts in the MTP reaction within the English literature. It is hoped that this work will offer valuable insights for advancing the development of catalytic systems in methanol conversion processes.

## 1. Introduction

Nowadays, there is great attention to the development of new supports in different methanol conversion processes. Alumina supports have gained widespread use as catalysts in the petroleum industry [1–6], attributed to their distinct characteristics, including high surface area, large pore volume, excellent thermal stability and high chemical activity [7–9].

According to the literature reports [10,11], expensive precursors and surfactants have been commonly used in the alumina synthesis pathway. The importance of the presence of surfactant in these synthesis methods is due to the fact that the surfactant has the ability to assemble with inorganic species to form mesostructured pore structures. Additionally, the use of different surfactants can result in the formation of distinct microstructures, highlighting the importance of selecting the appropriate surfactant for a desired pore structure [12].

Among various transitional phases of aluminium oxides [13–15], mesoporous γ-Al_2_O_3_ plays an important role in methanol dehydration to dimethyl ether (DME) [16,17]. DME is not only utilized as a final product but also serves as a precursor for the production of various chemical compounds [18,19]. With the aid of zeolite catalysts, DME can be transformed into light olefins and aromatics [20,21].

Importantly, the methanol-to-propylene (MTP) process is a pathway for converting methanol into hydrocarbons, which can be catalysed by microporous zeolites owing to their significant structure and the increased selectivity of the corresponding conversion towards olefins [22–26].

The MTP reaction process is of great interest owing to the finite nature of oil resources [27,28]. It is worth noting that this process typically begins with the dehydration of methanol to dimethyl ether, resulting in the formation of an equilibrium mixture comprising methanol, dimethyl ether and water. In subsequent steps, this equilibrium mixture is transformed into light olefins, and the specific catalyst and process conditions play a significant role in determining the outcome of the reaction [29].

In the present work, a facile and practical process for obtaining nanosized γ-Al_2_O_3_ using a polyethylene glycol template with different molecular weights, which may reduce the cost, was introduced. Since, as mentioned above, the suggested intermediate in the MTP conversion mechanism is dimethyl ether, with the aim of improving the catalytic activity of the H-ZSM-5 zeolite catalyst, the prepared AlPEG20000 was added to it, and this combination was used in two ways; physically mixed and unmixed (two-supported), in the MTP reaction process.

## 2. Material and methods

### 2.1. Chemicals and Instruments

Polyethylene glycol with different molecular weights (200, 6000 and 20 000), aluminium nitrate (Al(NO_3_)_3_·9H_2_O), ammonia (25%), ammonium carbonate and ammonium bicarbonate were commercially purchased from Aldrich, Fluka or Merck and all solvents and compounds were utilized without further purification. Fourier transform infrared (FT-IR) spectra were recorded using an ABB Bommem MB-100 FT-IR spectrophotometer with KBr pellets. X-ray diffraction (XRD) patterns were obtained using a D4 ENDEAVOR diffractometer with Cu Kα radiation source. Transmission electron microscopy (TEM) analysis was conducted using a Philips EM 208S microscope. The specific surface area was determined using the Brunauer–Emmett–Teller (BET) method, and the pore volume and pore size distribution were calculated by the Barrett–Joyner–Halenda method.

### 2.2. Synthesis of AlP20000

In the first step, 50 ml of aluminium nitrate solution (A) with a concentration of 1 mol l^−1^ and 50 ml of ammonium carbonate or ammonium bicarbonate solution (B) with a concentration of 1.75 mol l^−1^ were prepared using deionized water. Next, 15 g of polyethylene glycol (molecular weight 20 000) was added to the aluminium nitrate solution and stirred until completely dissolved. This resulted in a polyethylene glycol solution with a concentration of 0.15 mol l^−1^.

In a typical reaction, solution A containing polyethylene glycol and solution B were mixed dropwise simultaneously at room temperature while using a magnetic stirrer (250 rpm). Ammonia was added to the reaction mixture to increase the pH to 10. After ageing for about 20 h at room temperature, the prepared gel was transferred to a Teflon-lined stainless-steel autoclave and placed in an oven at a temperature range of 100–150°C. After 24 h the autoclave was cooled to room temperature. The resulting gel was then filtered, washed several times with distilled water and dried overnight at 80°C. Finally, the resulting powder, which was the synthesized pseudo-boehmite product, was calcined at 550°C for 4 h to produce γ-alumina.

### 2.3. Reactor test

The catalytic activity of the prepared catalysts was evaluated using a fixed-bed continuous flow reactor with a weight hourly space velocity (WHSV) = 2.3 h^−1^, in the presence of nitrogen as a carrier gas. Methanol conversion and product selectivity were monitored using a Varian 3800 RGA gas chromatograph.

## 3. Results and discussion

### 3.1. Preparation and characterization of γ-alumina samples

**Scheme 1.**
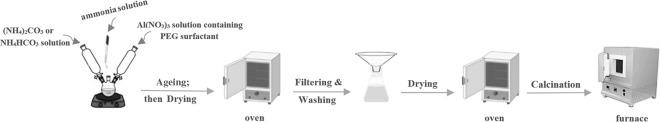
γ-Al_2_O_3_ synthesized through a step-by-step process.

Mesoporous alumina synthesis and characterization, with the aim of increased surface area, pore volume and suitable pore size distribution, have been reported. As illustrated in scheme 1, mesoporous γ-aluminas were synthesized via a few consecutive steps, using combination of sol–gel and hydrothermal methods. In this synthesis, each of the compounds aluminium nitrate, polyethylene glycol and ammonium carbonate or ammonium bicarbonate were used as precursor, surfactant and precipitating agent, respectively.

First, aluminium nitrate solution containing PEG surfactant and ammonium carbonate or bicarbonate solutions were mixed and stirred at room temperature. Subsequently, ammonia solution was used to adjust the pH. Afterwards, an ageing step was carried out and then the gel was placed in an autoclave. The final product (γ-Al_2_O_3_) was obtained after calcining the dried sample. The calcined samples prepared in the presence of (NH_4_)_2_CO_3_ and NH_4_HCO_3_ are labelled AlCP and AlBP, respectively, in this article. It should be noted that, in each synthesis, a known amount of polyethylene glycol with different molecular weights (200, 6000 and 20 000) was used. Therefore, the compounds obtained are coded AlCP200, AlCP6000, AlCP20000, AlBP200, AlBP6000 and AlBP20000.

**Figure 1.**
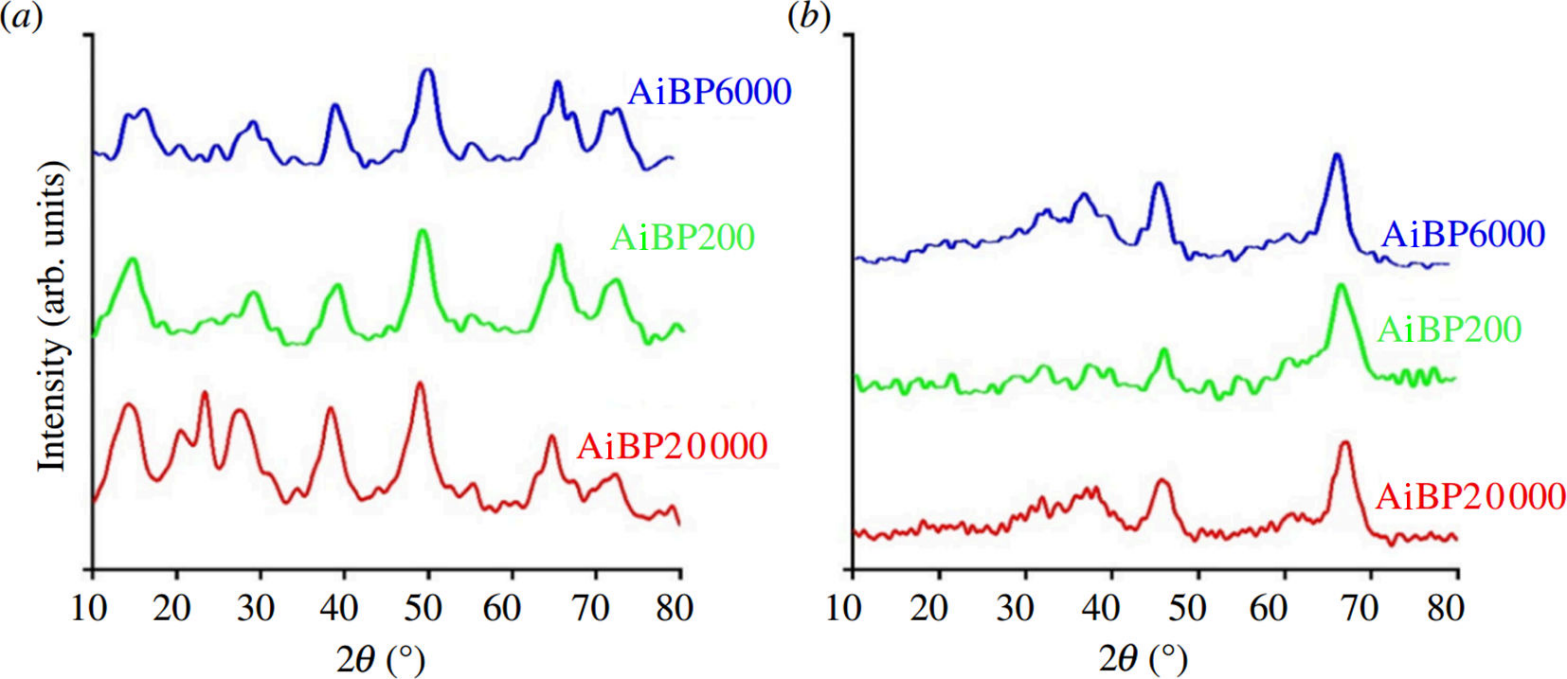
X-ray diffraction diagrams for samples using ammonium bicarbonate. (*a*) Boehmite and (*b*) γ-Al_2_O_3_.

**Figure 2.**
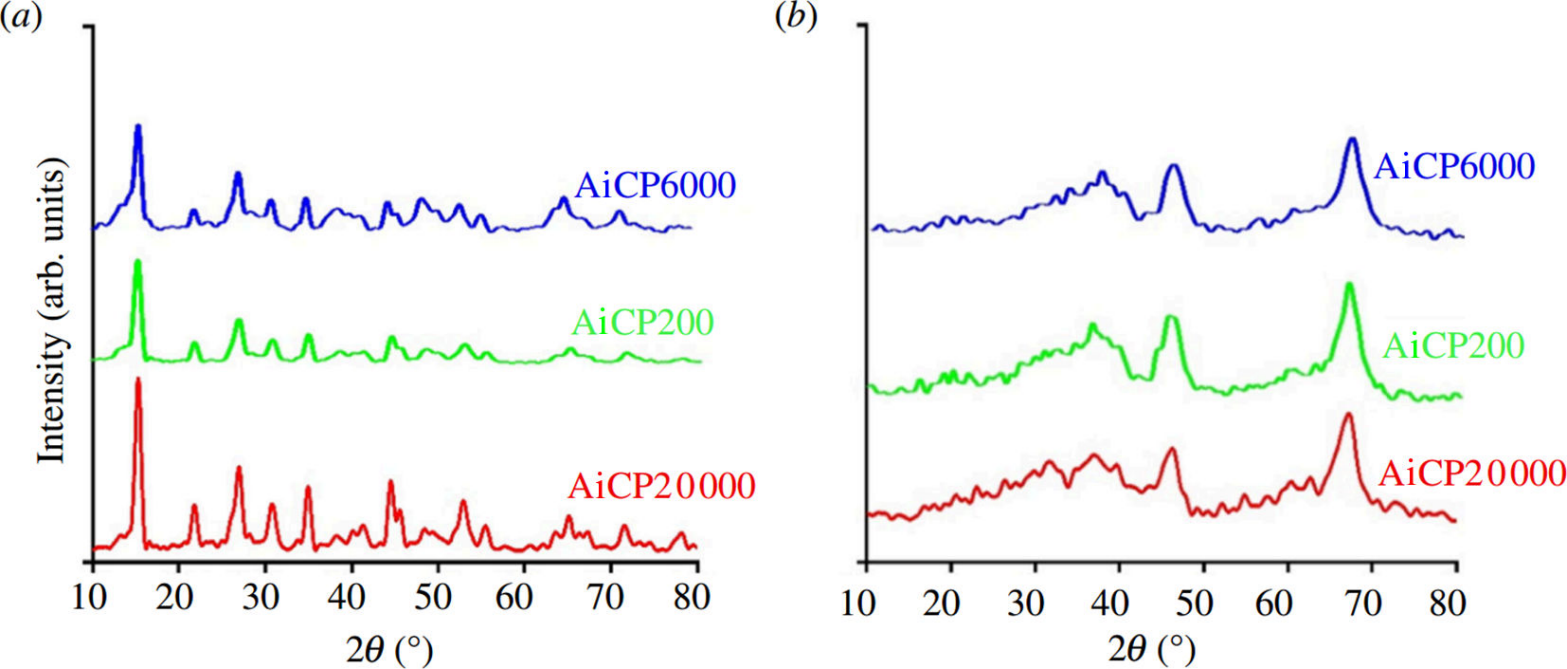
X-ray diffraction diagrams samples using ammonium carbonate. (*a*) Ammonium aluminium carbonate hydroxide and (*b*) γ-Al_2_O_3_.

Figures 1 and 2 display the XRD diffraction peaks of all samples. The diffraction peaks of the calcined products correspond perfectly with those of standard pure γ-alumina without impurity phases. These diffraction peaks are consistent with the database in ICDD file no. 10-425 [10]. Furthermore, in the XRD pattern shown in figure 2*a*, the characteristic peaks of ammonium aluminium carbonate hydroxide (JCPDS card no. 42-0250) are clearly observed [30].

**Figure 3.**
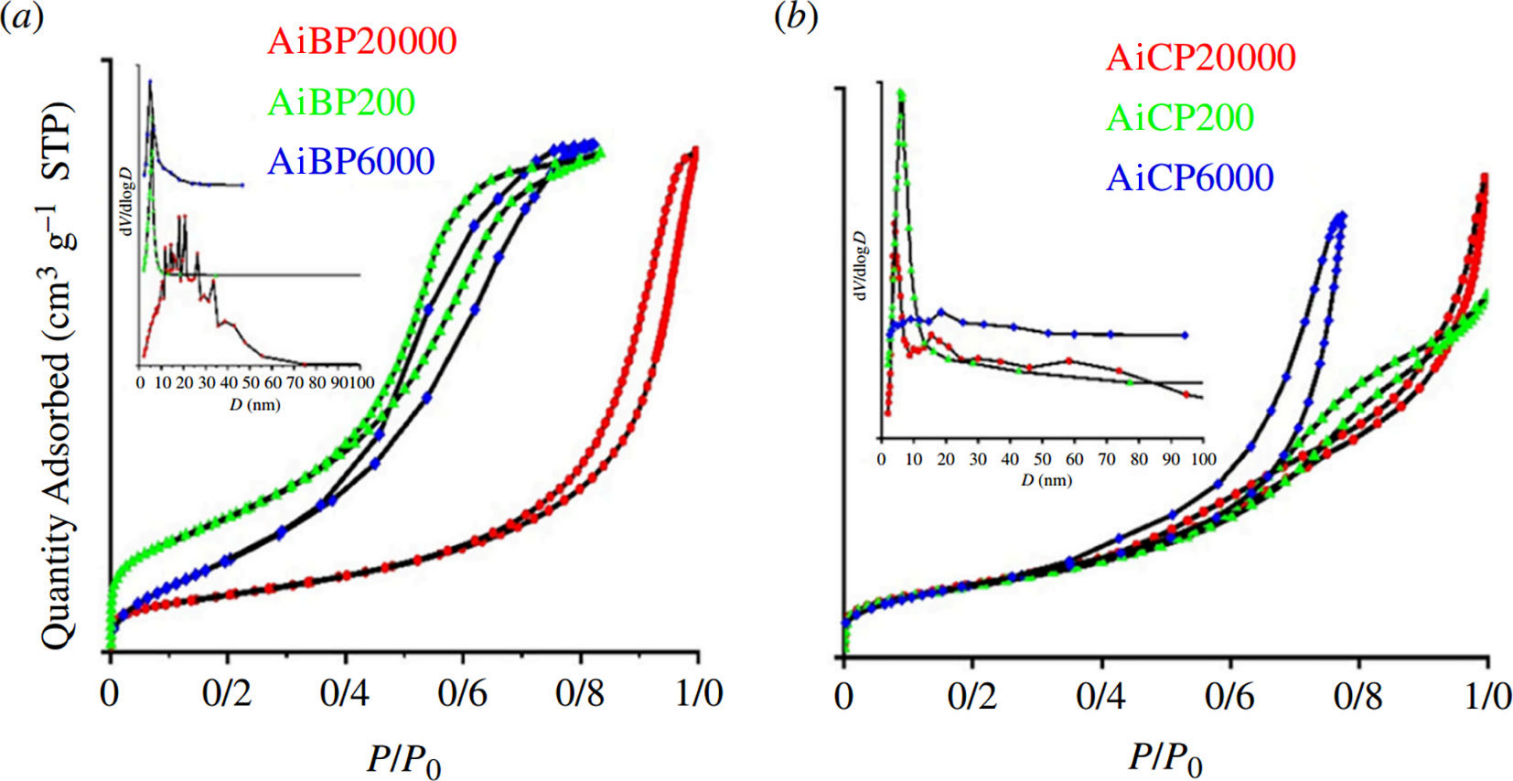
Nitrogen adsorption–desorption isotherms and pore size distribution of (*a*) samples synthesized in the presence of ammonium bicarbonate (*b*) samples synthesized in the presence of ammonium carbonate. Calcination conditions: 550°C, 4 h.

The nitrogen adsorption–desorption isotherms for the γ-Al_2_O_3_ samples are presented in figure 3. According to the IUPAC classification [31], the isotherms in figure 3*a* show type IV with H1-type hysteresis loop for AlBP20000 and H2-type hysteresis loop for AlBP200 and AlBP6000. Furthermore, figure 3*b* represents type IV with H1-type hysteresis loop for AlCP6000 and AlCP20000, and also H2-type hysteresis loop for AlCP200.

BET analysis was conducted on the γ-Al_2_O_3_ samples to determine their surface areas from nitrogen adsorption isotherms. The material parameters of all products obtained from the nitrogen adsorption–desorption study are summarized in table 1. The surface area of the samples ranged from 226 to 371 m^2^ g^−1^, and the pore volume varied from 0.45 to 1.26 cm^3^ g^−1^. The results indicate that using polyethylene glycol with a high molecular weight tends to increase the pore volume and surface area. The interaction of PEG molecules with other compounds in the reaction mixture was observed to significantly improve the textural properties of the alumina support.

**Table 1.** Textural properties of γ-Al_2_O_3_ samples.

sample	BET surface area (m^2^ g^−1^)	pore volume (cm^3^ g^−1^)	pore size (nm)
AlBP20000	349	1.26	14.4
AlBP6000	257	0.46	7.2
AlBP200	278	0.45	6.5
ALCP20000	232	0.59	10.1
ALCP6000	253	0.58	9.1
ALCP200	226	0.47	8.4

**Figure 4.**
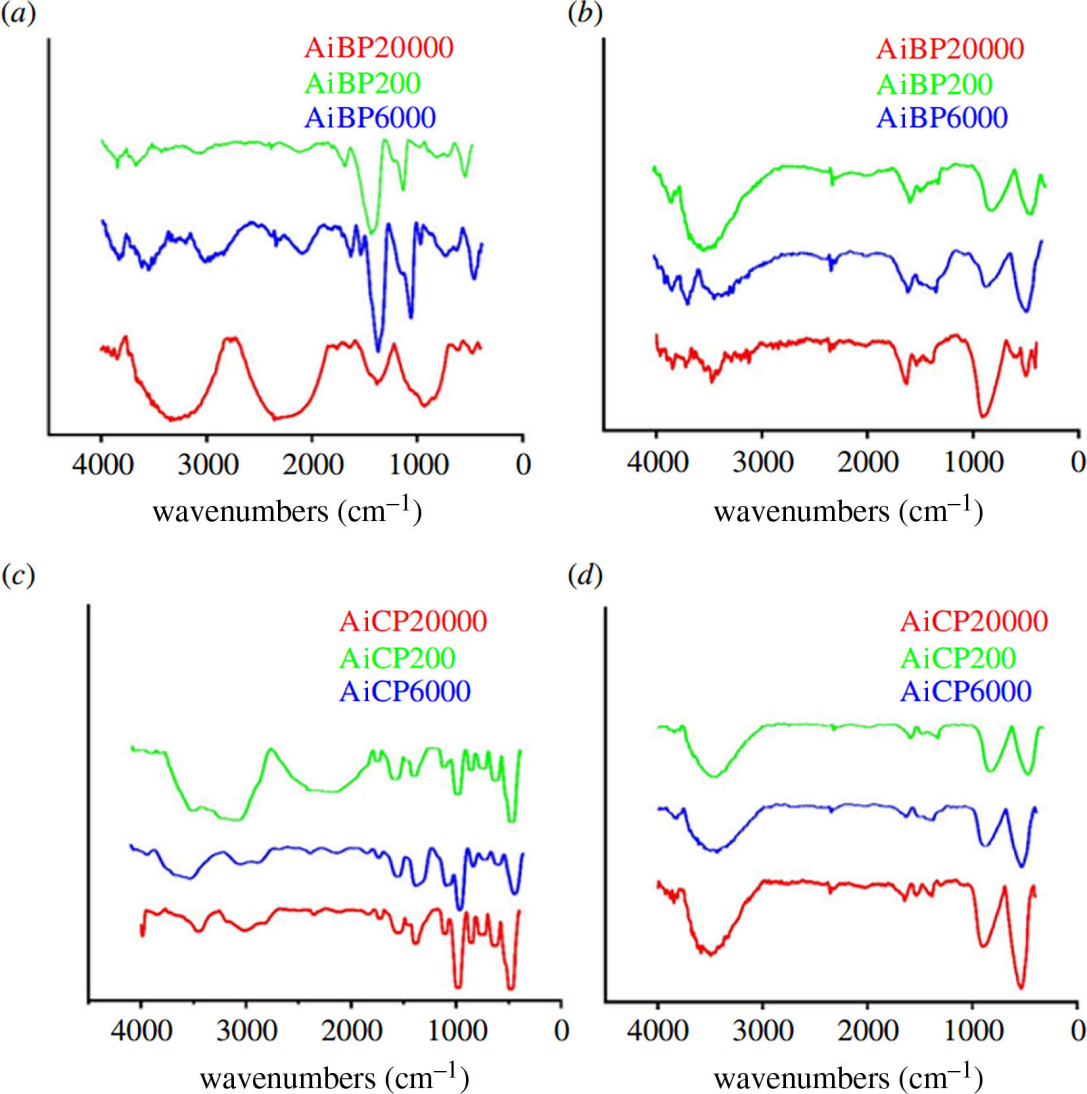
Fourier transform infrared spectra of (*a*) boehmite, (*b*) calcination of boehmite, (*c*) ammonium aluminium carbonate hydroxide and (*d*) calcination of ammonium aluminium carbonate hydroxide. Calcination conditions: 550°C, 4 h.

The FT-IR spectrum in figure 4 shows broad bands at around 3500 and 1630 cm^−1^, corresponding to the stretching and bending vibration modes of water molecules. The absorption bands in the range 400–1000 cm^−1^ were assigned to the stretching vibration of Al–O bonds [32]. The Al–O stretching modes of [A1O_6_] were observed below 900 cm^−1^ (898, 618, 544 and 414 cm^−1^) [33,34].

The symmetrical Al–OH bending modes were attributed to the band at 1069 cm^−1^, confirming the formation of boehmite. The calcined alumina free of PEG was confirmed by the elimination of the C–H band around 1384 cm^−1^. The bands around 500–750 cm^−1^ were associated with ν-AlO_6_, while the band at 900 cm^−1^ corresponded to ν-AlO_4_ [34,35]. The bands from 1400 to 1600 cm^−1^ clearly indicate the formation of aluminium, and their intensity gradually decreased after calcination, which was related to the rapid growth of the crystalline nanoparticles.

**Figure 5.**
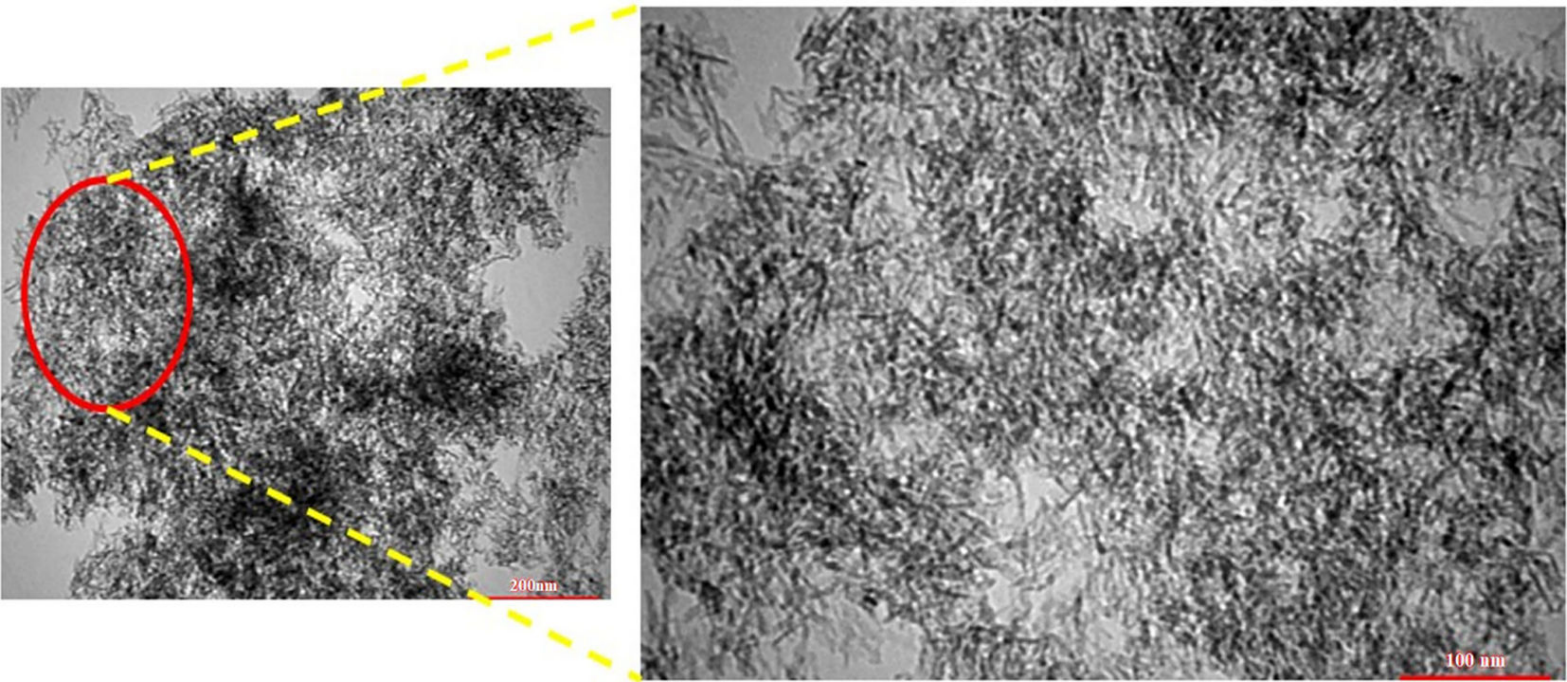
Tramsmission electron microscopy images of AlBP20000 sample after calcination at 550°C for 4 h.

The AlBP20000 sample, which had the highest pour volume and surface area, was chosen for further investigation. TEM was utilized to investigate the morphology of the synthesized alumina. The TEM images of the AlBP20000 sample (figure 5) clearly depict a wormhole-like morphology, as expected based on other similar studies, and confirm mesoporosity of the sample, as seen by BET analysis [36,37].

**Figure 6.**
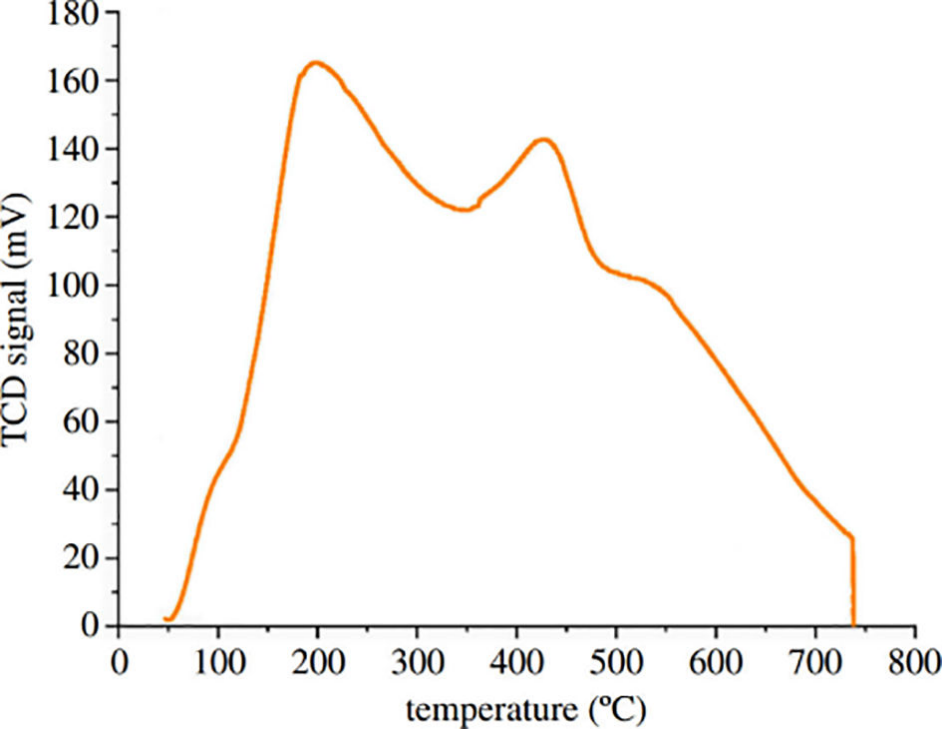
Ammonia temperature programmed desorption profile of synthesized alumina catalyst (AlBP20000). TCD, thermal conductivity detector.

Ammonia temperature programmed desorption (NH_3_-TPD) analysis was carried out to investigate the distribution of acidic sites on the γ-aluminas, as shown in figure 6. The surface of the synthesized AlBP20000 sample contains mainly three types of acidic sites: weak (160–350°C), medium (280–490°C) and strong (490–570°C). The low intensity of the peak related to strong acid sites in this sample suggests a low density of strong acid sites in the AlBP20000 catalyst. Furthermore, the NH_3_-TPD profile indicates that the total amount of acidic sites in the γ-alumina catalyst is 1.78 mmol NH_3_ g^−1^ catalyst.

### 3.2. Catalytic activity

**Figure 7.**
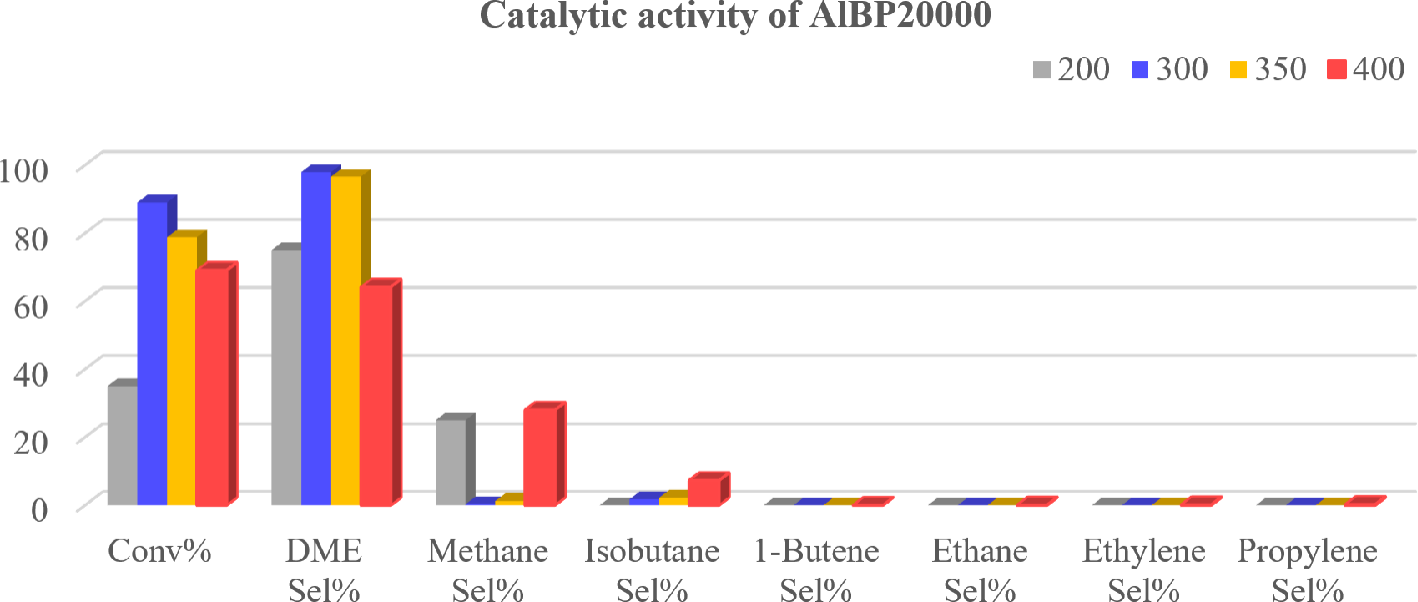
Methanol conversion (Conv%) and selectivity (Sel%) of gaseous products in the presence of the synthesized AlBP20000 catalyst at different temperatures (*T* = 200, 300, 350 and 400°C).

First, the catalytic performance of the AlBP20000 catalyst in the methanol-to-DME reaction was investigated (figure 7). Methanol conversion and selectivity towards DME were studied from 200 to 400°C. It was found that the optimum temperature for the reaction in the presence of the synthesized alumina catalyst prepared using PEG20000 and bicarbonate, AlBP20000, was between 300 and 350°C. At this temperature range, the catalyst demonstrated 98% selectivity towards DME and 89% conversion of methanol.

It should be mentioned that the synthesized alumina AlBP20000 catalyst exhibits high catalytic activity and efficiency due to its good structural properties such as high surface area and pore volume (as observed in table 1).

**Figure 8.**
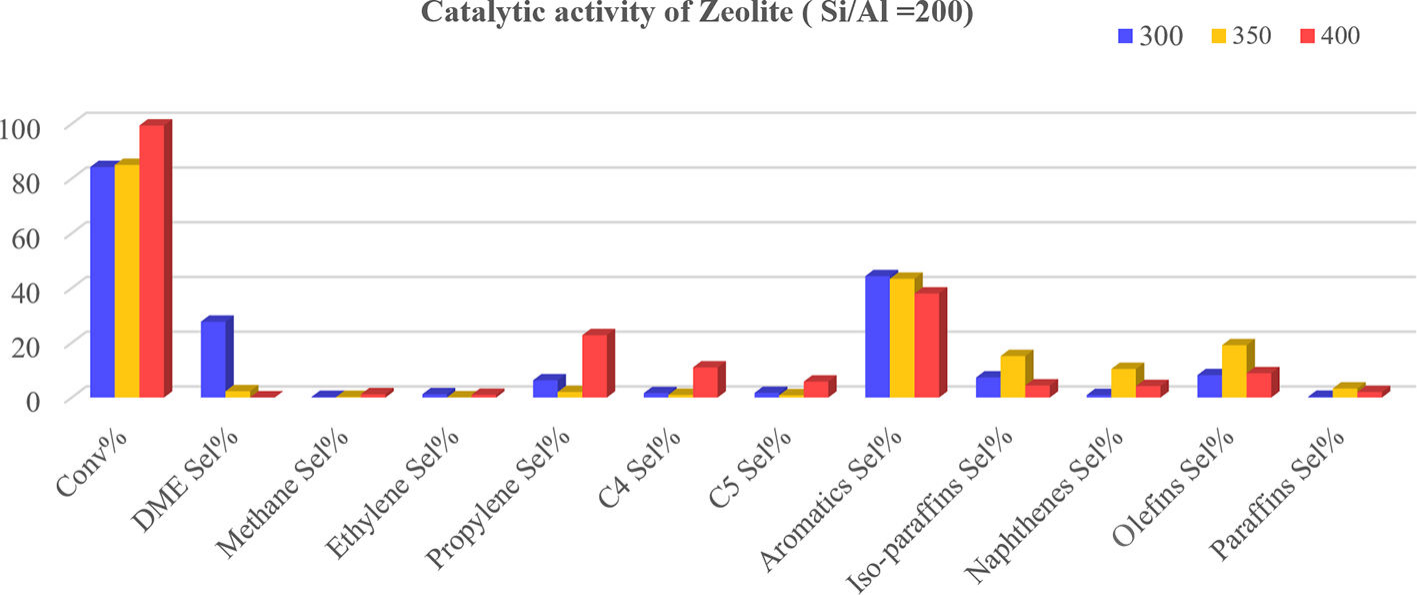
Methanol conversion (Conv%) and selectivity (Sel%) of gaseous and liquid products in the presence of zeolite catalyst (H-ZSM-5) at different temperatures (*T =* 300, 350 and 400°C).

**Figure 9.**
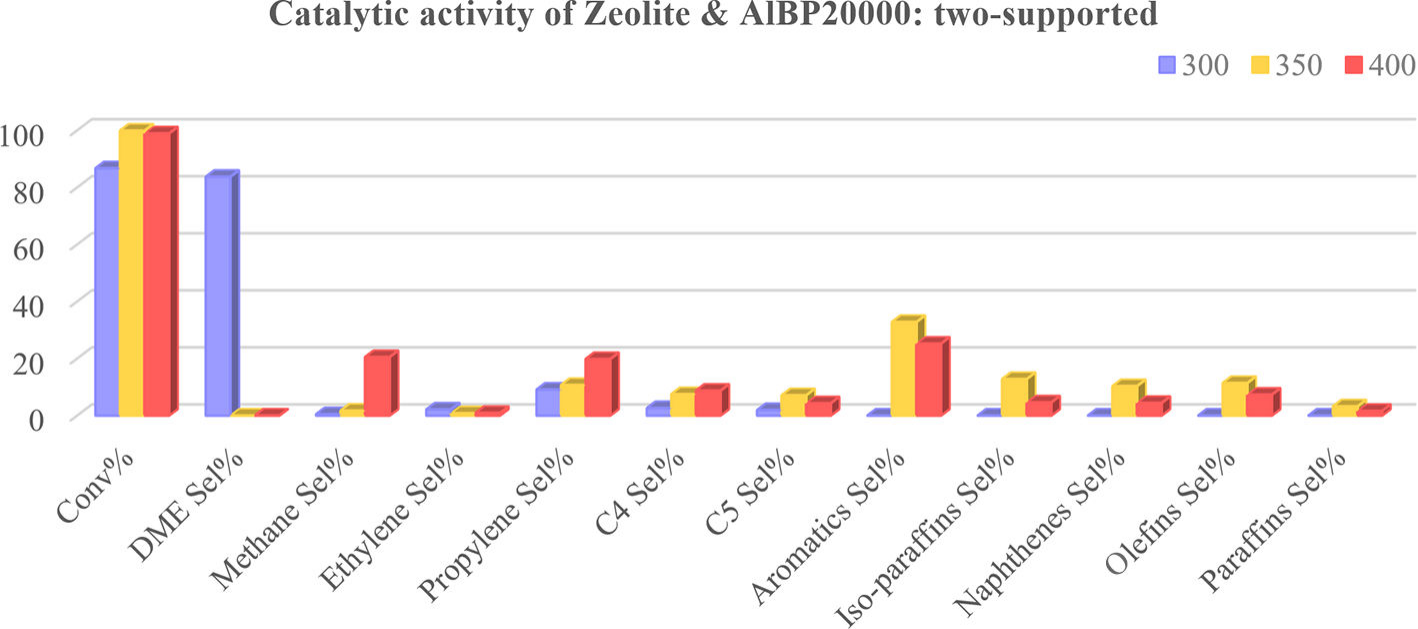
Methanol conversion (Conv%) and selectivity (Sel%) of gaseous and liquid products in the presence of physically unmixed synthesized AlBP20000 plus zeolite at different temperatures (*T* = 300, 350 and 400°C).

**Figure 10.**
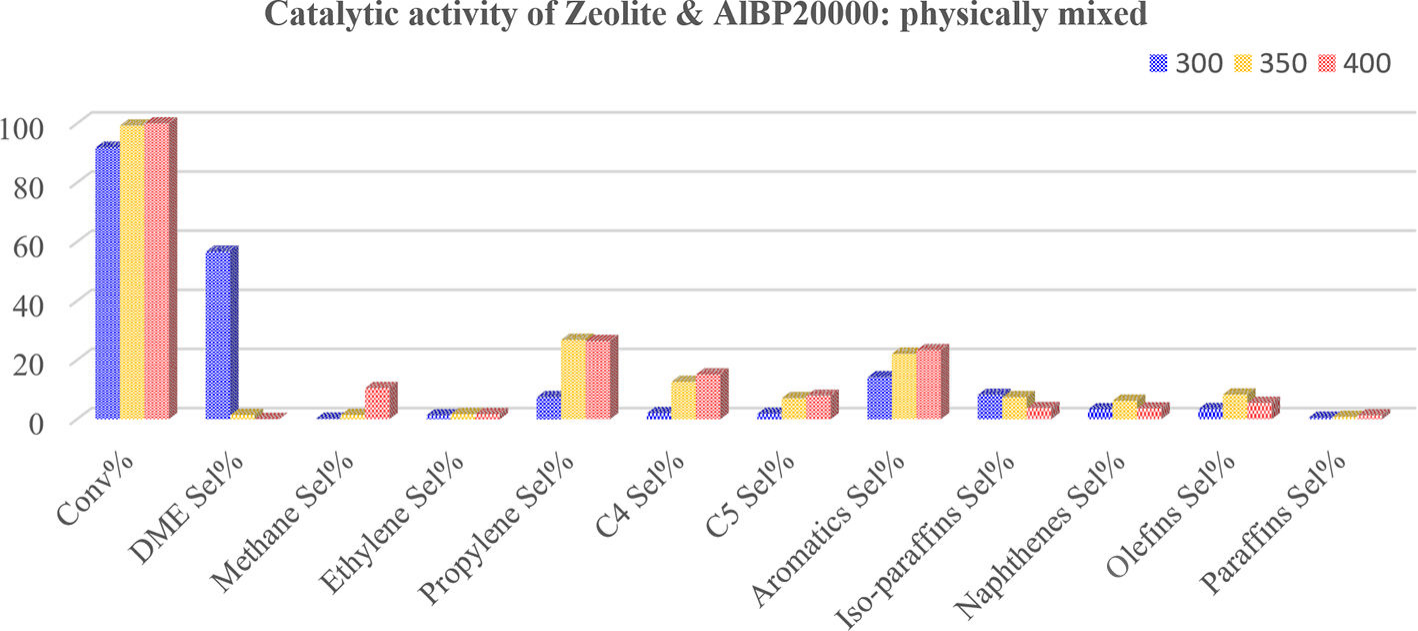
Methanol conversion (Conv%) and selectivity (Sel%) of gaseous and liquid products in the presence of physically mixed synthesized AlBP20000 plus zeolite at different temperatures (*T =* 300C, 350 and 400°C).

Afterwards, the catalytic activity of commercial H-ZSM-5 zeolite in the methanol-to-propylene reaction and the effect of the presence of physically mixed and unmixed (two-supported) synthesized AlBP20000 with the zeolite, to improve the performance of the zeolite, were studied( figures 8–10).

It is important to note that the reason for investigating the catalytic performance of the AlBP20000 sample with zeolite is its favourable structural properties compared with other synthesized γ-aluminas. The results obtained from the study revealed that methanol conversion was generally raised by temperature for all three prepared catalysts, as shown in figures 8–10, indicating the strong influence of temperature on the reaction. As the temperature increases, other trends are that the selectivity for DME production decreases and the selectivity towards propylene increases.

It must be noted that in the methanol-to-propylene process, methanol is initially dehydrated to DME, resulting in a mixture containing methanol, DME and water. This equilibrium mixture is then converted into light olefins, which can be further processed catalytically in the last step to produce a variety of products, including aromatics, paraffins, naphthenes and higher olefins via hydrogen transfer, alkylation or polycondensation reactions [32]. As illustrated in figures 9 and 10, the selectivity for DME production increases at 300°C with the presence of γ-Al_2_O_3_ in the catalyst.

The methanol-to-propylene (MTP) reaction mechanism in the presence of a physically mixed mesoporous alumina with H-ZSM-5 zeolite catalytic system can be described in two main steps: the dehydration of methanol to DME on γ-Al_2_O_3_ and the transformation of DME to light olefins on H-ZSM-5. In the first step, methanol adsorbs onto the γ-Al_2_O_3_ surface, where it interacts with acidic sites and undergoes dehydration to form DME. The DME and water are then desorbed from the catalyst surface. In the second step, DME adsorbs onto the H-ZSM-5 zeolite. Through a series of cracking and methylation reactions, hydrocarbon pool intermediates are formed. These intermediates further react to produce light olefins, which are then desorbed from the zeolite surface.

**Figure 11.**
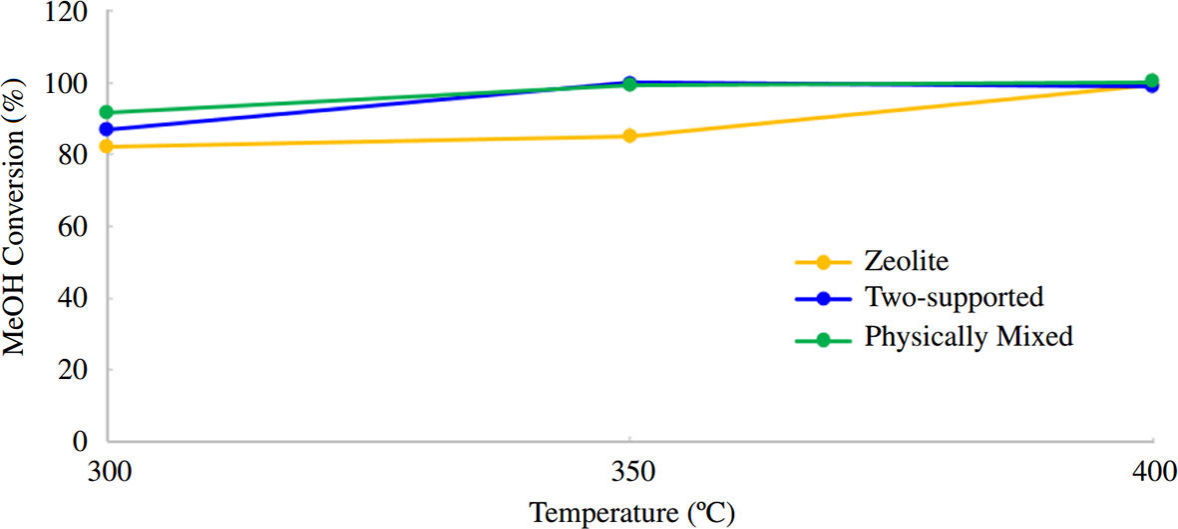
Comparison of the catalytic activity of the prepared catalysts in terms of methanol conversion at different temperatures (*T =* 300, 350 and 400°C).

**Figure 12.**
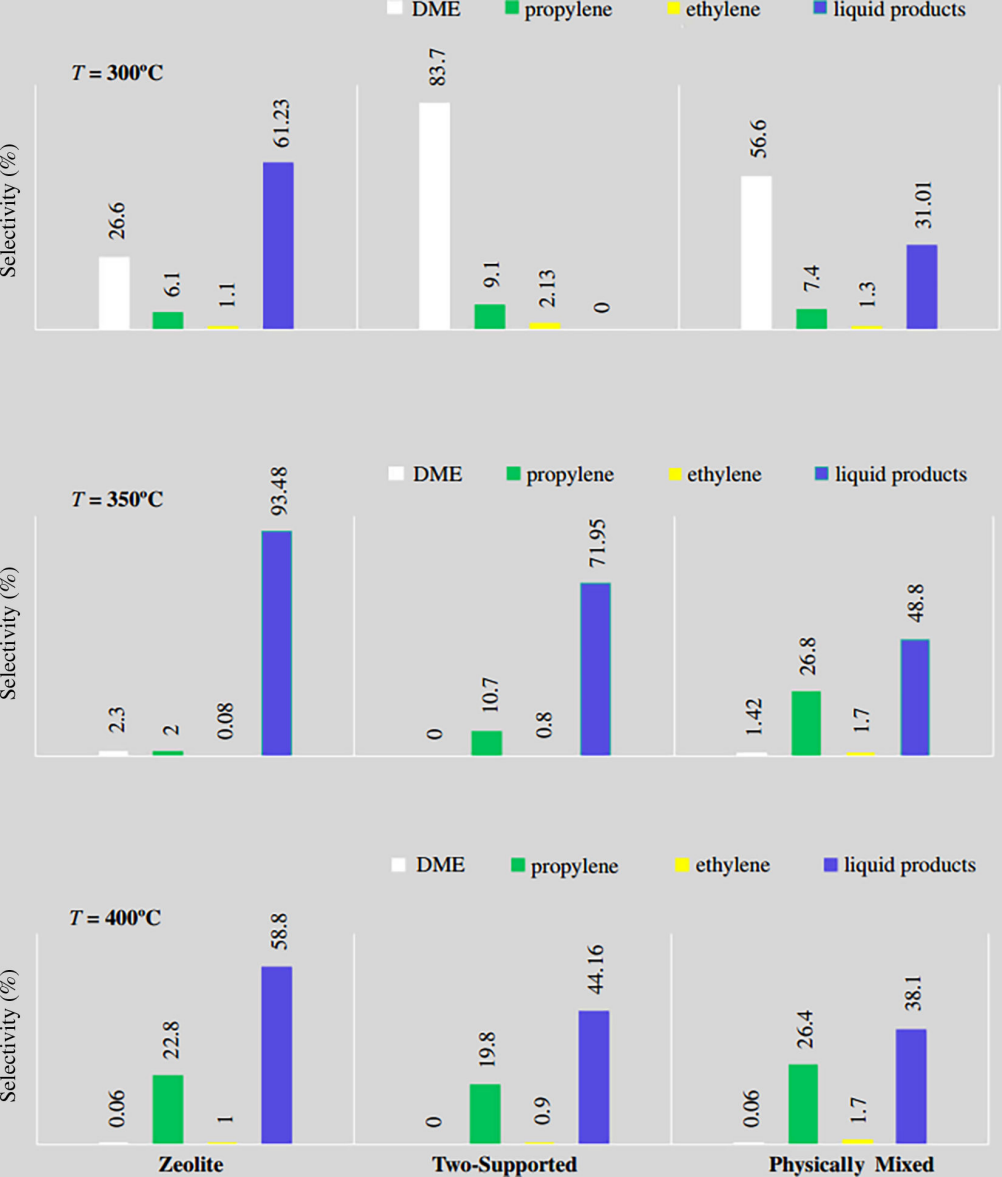
Comparison of the catalytic activity of the prepared catalysts in terms of selectivity at different temperatures (*T =* 300, 350 and 400°C). DME, dimethyl ether.

Finally, the catalytic activity of the prepared catalysts were compared in terms of methanol conversion and propylene selectivity at different temperatures after 2 h, as illustrated in figures 11 and 12, respectively. After conducting reactor tests, it was determined that the optimum reaction temperature is 400°C, as this temperature yielded the highest propylene selectivity and methanol conversion rates.

**Figure 13.**
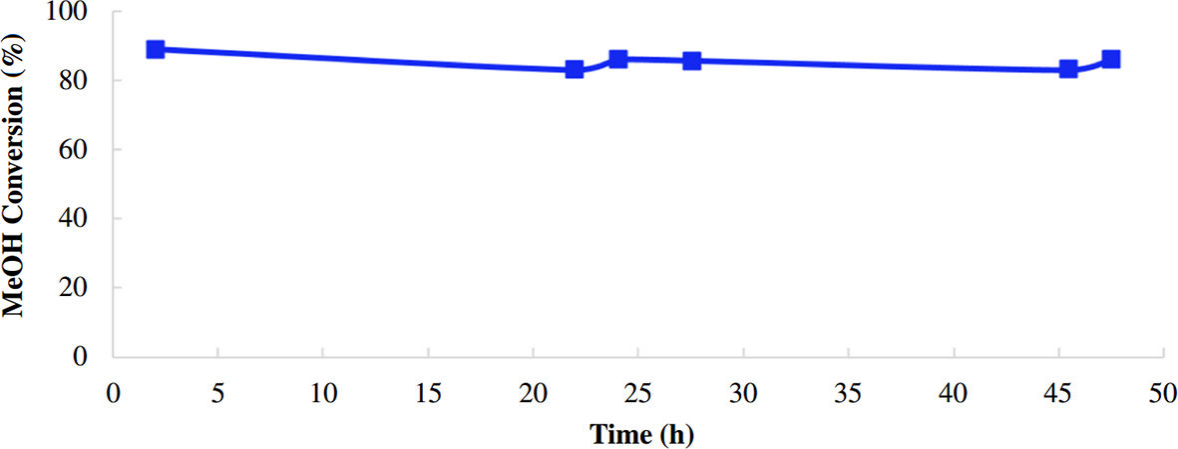
Time-on-stream of the synthesized alumina catalyst (AlBP20000) at 300°C, *p* = 1 atm, weight hourly space velocity (WHSV) = 2.3 h^−1^.

The time-on-stream of the prepared catalyst (AlBP20000) was studied to show its stability over time (figure 13). The results obtained demonstrate excellent stability of the catalyst over a period of 48 h and methanol conversion remains almost constant during this time.

**Figure 14.**
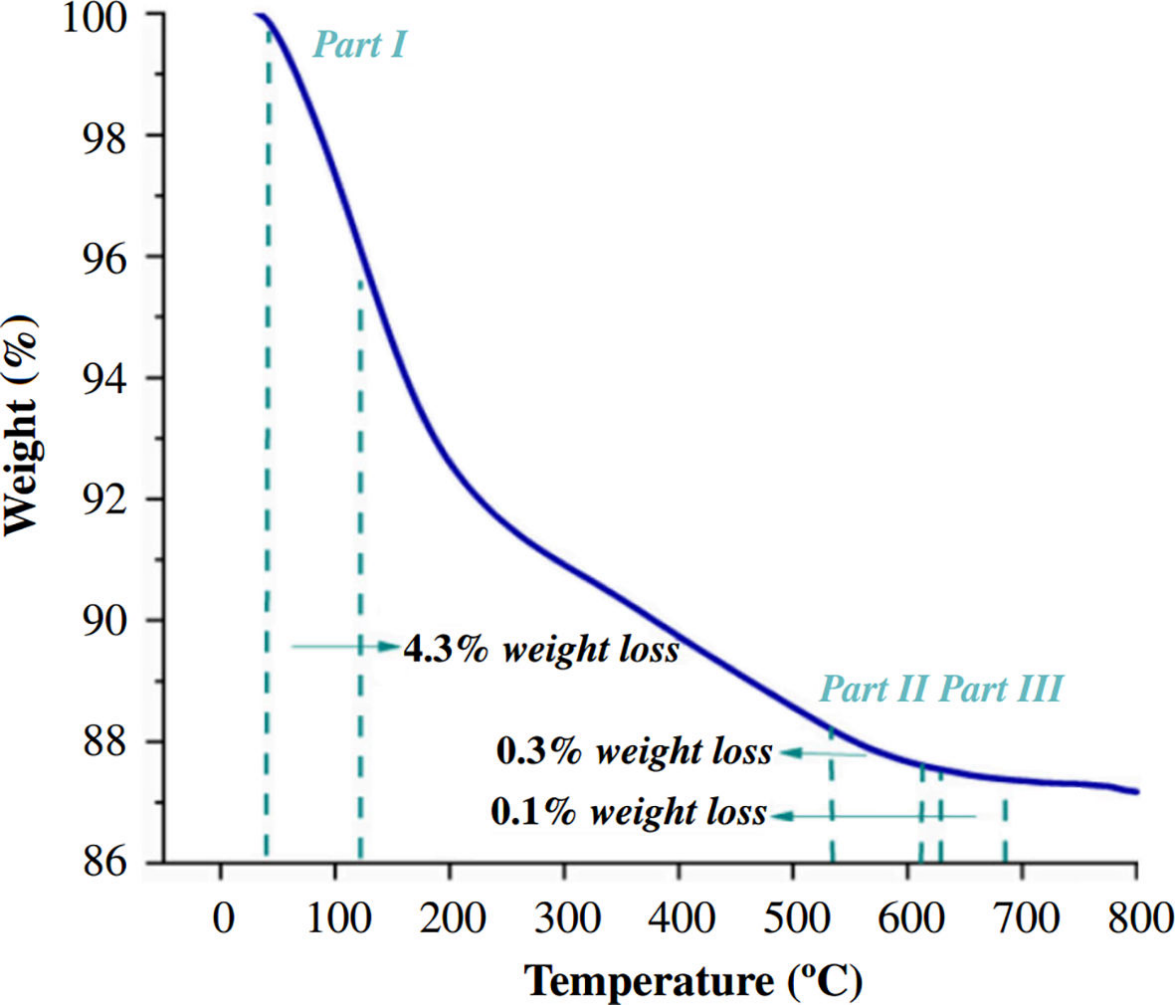
Thermogravimetric analysis diagram of the used AlBP20000 catalyst after 48 h.

To evaluate the stability of the catalyst, TGA was performed on the used AlBP20000 catalyst at the conclusion of the reactor test reaction process. The resulting TGA diagram shows weight loss in three distinct temperature ranges (figure 14). The first temperature range corresponds to the loss of adsorbed water, while the second and third temperature ranges are associated with surface coke combustion and heavy and encapsulated coke combustion, respectively.

## 4. Conclusions

Facile structure-controlled synthesis of γ-aluminas with high surface area using polyethylene glycol templates with different molecular weights was studied in this work. Also, catalytic activity of one of these synthesized aluminas, AlBP20000 (owing to its good structural properties), and commercial H-ZSM-5 zeolite catalyst was investigated in two ways—physically mixed and unmixed—in the MTP reaction. In other words, the performance of H-ZSM-5 zeolite in the presence of the synthesized alumina, with the aim of improving the catalytic activity of the zeolite, is reported.

The textural properties of the alumina-PEG20000 support, which can be tailored by adding a surfactant, could affect its catalytic performance. A notable outcome of this study is the remarkable activity demonstrated by the synthesized catalysts in both physically mixed and unmixed states during the MTP process at 400°C, as evidenced by high rates of methanol conversion and by propylene selectivity.

The enhanced catalytic activity and improved selectivity towards propylene in the MTP process can be attributed to several synergistic effects between the alumina and H-ZSM-5 components. γ-Al_2_O_3_ is highly efficient in catalysing the dehydration of methanol to DME, an essential intermediate in the MTP process. The presence of γ-Al_2_O_3_ increases the concentration of DME available for subsequent conversion on H-ZSM-5, thereby enhancing the overall reaction rate. Additionally, the acidic sites on γ-Al_2_O_3_ complement those on H-ZSM-5, creating a more effective bifunctional catalyst system. This combined acidity facilitates the initial dehydration of methanol and its subsequent conversion to propylene, enhancing overall catalytic activity and selectivity. As a result, these synergistic effects between synthesized mesoporous alumina (AlBP20000) and H-ZSM-5, including enhanced methanol dehydration and increased acidity, significantly contribute to the improved MTP conversion and selectivity observed in this study.

## Data Availability

This article has no additional data. All data are reported in the main body of the article. Datasets have been cited in the reference list.
